# The Importance of Dispersal for Bacterial Community Composition and Functioning

**DOI:** 10.1371/journal.pone.0025883

**Published:** 2011-10-06

**Authors:** Eva S. Lindström, Örjan Östman

**Affiliations:** 1 Department of Ecology and Genetics/Limnology, Uppsala University, Uppsala, Sweden; 2 Department of Ecology and Genetics/Population biology, Uppsala University, Uppsala, Sweden; Netherlands Institute of Ecology, Netherlands

## Abstract

We conducted a metacommunity experiment to investigate the role of dispersal for bacterial community composition (BCC) and function of freshwater bacteria. Bacteria were dispersed from a common source pool into three different lake communities in their natural lake water. The experiment was conducted in dialysis bags to enable a decoupling between a change in the local environment and dispersal. BCC was determined by terminal restriction fragment length polymorphism (tRFLP) of the 16S rRNA gene. We show that the greatest changes in BCC occurred between 10% and 43% of dispersal of standing stock per day. Functioning, measured as growth rate, was also affected by dispersal in all three communities but the qualitative pattern differed between communities, sometimes showing a hump-shaped relationship to dispersal and sometimes decreasing with increasing dispersal. In all waters, functioning was related to BCC. Our results show that dispersal does affect BCC and functioning but that high dispersal rates are needed. Further, the effect of dispersal on BCC and function seem to depend on the quality of the habitat to which bacteria disperse into.

## Introduction

Due to the concern of which consequences the loss of biodiversity will have on ecosystems, it is of considerable interest to evaluate the importance of biodiversity for ecosystem functions (e.g. [1], [2]). The mechanisms by which communities assemble may be of importance for ecosystem functioning, since different diversity-productivity patterns can be obtained depending on whether communities are assembled through local (niche) or regional (dispersal) processes [3], [4]. If assembly via dispersal is important for communities, dispersal should have an immediate effect on the composition. The functioning, on the other hand, should, in theory, increase with dispersal because of the inclusions of taxa with complementary traits. However, at high dispersal rates we may also expect a decrease in functioning because of a dilution of locally adapted taxa [5]. In contrast, if local niche processes are most important for the assembly of communities, dispersal should insignificantly affect community composition and function [5].

In an experimental study, Venail et al. [6] showed that bacterial growth at the regional scale was maximized at intermediate dispersal in an evolving metacommunity because of high niche differentiations among strains. Further, in experimentally unsaturated benthic microalgae communities, [7], [8] a hump-shaped relationship between dispersal and biomass was found. Thus, there is empirical support of a dependence of function on dispersal, although the number of studies is low. In addition, dispersal manipulations of natural communities in order to explain functional patterns in relation to dispersal are missing entirely, and therefore we here aim to experimentally study dispersal effects on natural bacterial communities.

Bacterial communities can serve as practical models in ecology since experiments and field studies can relatively easy (in comparison to larger organisms) be designed over a range of relevant temporal and spatial scales [9]. Since samples of more or less natural complexity can be brought into the laboratory, these organisms should be ideal for experimental studies for instance of diversity-function relationships. Further, relevant functional variables are easy to identify in bacteria due to their central role in ecosystems, for instance in the carbon cycle, where they make otherwise inaccessible carbon accessible for the rest of the food web (e.g. [10]).

Results obtained from studies of diversity-functioning relationships in bacterial communities have so far yielded disparate results. If different bacteria play different roles in ecosystems, for instance regarding their ability to degrade different organic molecules, changes in community composition (BCC) should lead to an altered functionality (e.g. [11], [12], [13]). On the other hand, the wide diversity of bacteria indicates a functional redundancy among species, which weakens the link between community composition and functionality. In this case, the local conditions may instead be more important for differences in bacterial functioning (e.g. [14], [15]). Even though there is evidence for regional processes playing a role for BCC (e.g. [16], [17]), the results are varying regarding how high dispersal rates are needed to cause changes in the community composition (i.e. mass effects, [18], [19], [20], [21]). One complicating factor is that the passive migration of bacteria may be accompanied by a change in the local environment, e.g. high water flows also change organic matter quality [22], which may again influence function as well as community composition. Thus, for bacterial communities the relative importance of dispersal and species sorting dynamics (i.e. selection by the local environment; [23]) is under natural conditions often difficult to disentangle due to co-variation in statistical models. Therefore, empirical support of an effect of dispersal on community assembly and the relationship between community composition and function in natural bacterial communities is generally lacking.

In the present study we are conducting a laboratory experiment in which bacteria were dispersed into bacterial communities in dialysis bags kept in larger containers with natural water. Because the dialysis bags allow transportation of water and dissolved chemicals but not bacterial cells across the membrane, dispersal can be disconnected from a change in environmental conditions. The aim of this study was to evaluate 1) the magnitude of dispersal rates from a metacommunity that are needed to cause changes in local BCC, 2) whether dispersal from a metacommunity affects a functional trait, such as local bacterial growth rate, and 3) whether there is a relationship between BCC and the functional trait.

## Materials and Methods

### Experimental setup

We sampled water and the natural bacterial communities from three lakes with different characteristics to invoke large differences in environmental conditions between communities. No permits were required to sample these lakes. Lake Ånnsjön is an oligotrophic clear-water lake (total phosphorus (TP) = 15 µg L^−1^, total organic carbon (TOC) = 5 mg L^−1^, Absorbance at 430 nm, a measure of humic matter content, (Abs430) = 0.073), Lake Fälaren is a humic lake (TP = 21 µg L^−1^, TOC = 32 mg L^−1^, Abs430 = 0.172), and Lake Funbosjön is an eutrophic lake (TP = 62 µg L^−1^, TOC = 27 mg L^−1^, Abs430 = 0.131). Lakes Fälaren and Funbosjön were sampled on July 27 2009, and Lake Ånnsjön July 25 2009. The experiment started on the 29 of July 2009 prior to which all water was filtered through a 1 µm glass fibre filter (Gelman A/E) to remove larger organisms than bacteria.

From each lake we filled three 10 L buckets (replicates) with filtered water, i.e. in total there were nine buckets. In each bucket we placed five 10 mL dialysis bags (Float-A-Lyzer® G2, with a pore size of 20 000 Daltons, Spectrum®labs.com, USA) containing the natural bacterial community from that lake (one for each dispersal treatment). In addition we also added one dialysis bag with equal parts from the three different lakes (Mix) that served as a treatment with no continuous dispersal but where dispersal limitations between communities was eliminated. Thus, for the experiment we had 9*6 = 54 dialysis bags. In addition we also had one negative control dialysis bag of autoclaved MQ water in each bucket. The water in the buckets was mixed by vigorous air bubbling. The dialysis bags were pre-wetted in MQ water for one day before the initiation of the experiment.

Three times a day we dispersed bacteria into the communities in the dialysis bags by pipetting. At each dispersal event a metacommunity was first constructed from a mixture of equal volumes of the three original lake bacterial communities. That is, the three natural lake communities were kept in separate 1 L glass bottles under the same conditions as the experiment was run (see below). At dispersal, equal volumes were taken out from the three lake communities and mixed into a metacommunity which we used for dispersion of bacteria into the dialysis bags. See figure S1 for a graphical explanation of the experimental design.

Empirical estimates about how high dispersal rates are needed to affect bacterial communities in lakes are scarce, but there are indications that high dispersal rates are required [20]. Therefore, to make sure that we would record dispersal effects in our experiment, we chose to have a wide gradient of dispersal rates (as described above). The five different dispersal treatments were 0 (no dispersal), 0.033 mL, 0.33 mL, 1.7 mL, and 3.3 mL per dispersal event. In the three highest dispersal treatments an equally large volume was also pipetted out of the community in the dialysis bag before dispersing from the metacommunity. In the 0.033 mL and 0.33 mL the transferred bacteria into dialysis bags during one day corresponded to approximate 1% and 10%, respectively, of the standing bacterial abundance. For the two treatments with highest dispersal, dispersal rate was calculated as 1 minus the likelihood of not being transferred out from a dialysis bag during one day, i.e. 1-(1 – X/10)^3^, where X is volume transferred in and out of a dialysis bag per dispersal event. Thus, for the 1.7 mL and 3.3 mL treatments the approximate dispersal rates were 43% and 70% per day, respectively. The 0 dispersal and the “Mix” treatment received no dispersal during the course of the experiment, but were opened and closed to mimic the handling of the dispersal treatments.

In nature, lakes exist with water retention times of one day (i.e. 100% replacement per day), and in Sweden the great majority of lakes has a water retention time of less than 100 days [18], i.e. they have an in- and outflow of more than 1% per day. Thus, our dispersal rates are high but certainly relevant to natural lake bacterial communities.

The experiment was run for 5 days at 15°C in darkness. At the end of the experiment samples were taken from each dialysis bags for analyses of bacterial community structure, growth rate and abundance. Samples for community composition and abundance were also collected from the three start communities and the three communities making up the metacommunity from which dispersal occurred, at the end of the experiment.

Dialysis bags prevent movement of bacteria out of the bag but allow the local environmental conditions in the bags to be close to the conditions in the buckets, independent of dispersal. Compared to previous experiments on microbial communities (e.g. [24]) we were using dialysis bags of big pore sizes (20 kDa) to make the diffusion of molecules through the membranes as efficient as possible. In natural lake water the size of organic molecules varies greatly but the majority of dissolved organic molecules are smaller than 5 kDa ([25] and references therein). The short time duration of our experiment was chosen to avoid over-growth of the pores in the dialysis membranes, which would affect diffusion negatively. Still, the effective pore size can be expected to have been less than 20 kDa. To estimate how efficient organic matter diffused over the membrane, we measured the water colour (which should be affected by the content and composition of organic matter) as the absorbance at 430 nm, by the end of the experiment in the bucket water and in one replicate of dialyses bags with highest dispersal for each lake. The diffusion of inorganic matter through the membrane we measured in an extra bag filled with lake water and kept in the bucket with the most eutrophic lake water, i.e. Lake Funbosjön. On the last day of the experiment we emptied this bag of lake water and filled it with a solution of Na_2_HPO_4_ (1g L^−1^). The bag was put in a beaker with deionized water and we recorded conductivity in the beaker for up to 4 hours. The conductivity in this beaker was compared with the conductivity in a beaker where the same amount of salt had been added directly into the same amount of deionised water.

### Bacterial community structure

Bacterial community composition (BCC) was determined by terminal restriction fragment length polymorphism (tRFLP) of the 16S rRNA gene as described by Lindström et al. [19]. Briefly, the cells in the samples were collected by centrifuging 3 mL of water sample at 17 000 x g for 30 minutes in sterile Eppendorf tubes. First, 1.5 mL of sample were centrifuged and the supernatant discarded, and thereafter an additional 1.5 mL was added to the same tube and the procedure repeated. DNA was extracted and the 16S rRNA genes amplified by PCR.Digestion was carried out separately with the two restriction enzymes *HaeIII* and *HinfI*.

Peaks (tRFs) in lengths greater than 447 bp were eliminated from the analysis. Peaks with a relative area less than 0.5% were also eliminated from the analysis as these are difficult to separate from noise. The average relative peak area of restriction digest duplicates was used for further statistical analysis. In five samples, among which 4 were experimental negative controls (i.e. autoclaved MQ water), the amount of DNA extracted was too low to enable tRFLP analysis.

### Bacterial growth rate and abundance

1 mL samples for bacterial abundance were collected and preserved with 4% (final concentration) formaldehyde. Bacterial abundance was determined by flow cytometry (Partec) after staining with Syto13 at a final concentration of 1.25 µM [26]. Flow cytometry was run with forward scatter gain = 200 and threshold = 50, side scatter gain = 220 and threshold = 10, and fluorescence 1 gain = 450 and threshold = 10, triggered on fluorescence 1.

Bacterial growth rate was determined using the leucine incorporation method [27]. Tritiated leucine (15% labelled, 85% unlabelled) was added to 1.7 mL of each sample to a final concentration of 100 nM and incubated for 1 h in 15°C. One blank was created from the water in each bucket and treated in the same way as above except that the bacteria were killed with 5% TCA (final concentration) before the addition of leucine. The results are presented as disintegrations of tritium per minute (dpm) with the dpm of the blank from the respective bucket subtracted, and divided by bacterial abundance (dpm/cell).

### Statistics

Bray-Curtis (relative peak areas) as well as Sørensen (presence-absence of peaks >0.5%) dissimilarities [28] were calculated for all possible sample pairs and subsequently analysed by non-metric multidimensional scaling (NMDS) with the ‘isoMDS’-function in R 2.10.0. To study effects of dispersal treatments on community composition and if communities differed between lake waters we conducted a PerMANOVA on both Bray-Curtis and Sørensen dissimilarity matrices using the ‘adonis’-function in the ‘Vegan’-package. A PerMANOVA is like an ordinary ANOVA but partitions similarity matrices between treatments and uses permutation tests with pseudo F-ratios. We did a two-way ANOVA to study effects of dispersal treatments and lake waters on bacterial growth rates (dpm or dpm/cell as dependent variable). For each lake we did a one-way ANOVA of dispersal treatments on bacterial growth rates using Tukey's HSD for pairwise comparisons between dispersal treatments in R 2.10.0. To investigate if bacterial community composition covaried with bacterial growth rates, the NMDS sample scores were correlated to growth rate and specific growth rate by Spearman rank correlation analyses. When NMDS axes were used for correlation analyses, separate NMDS were run for each lake. Kruskal's stress values <0.15 for all lakes (2 axes).

## Results

**Figure 1:**
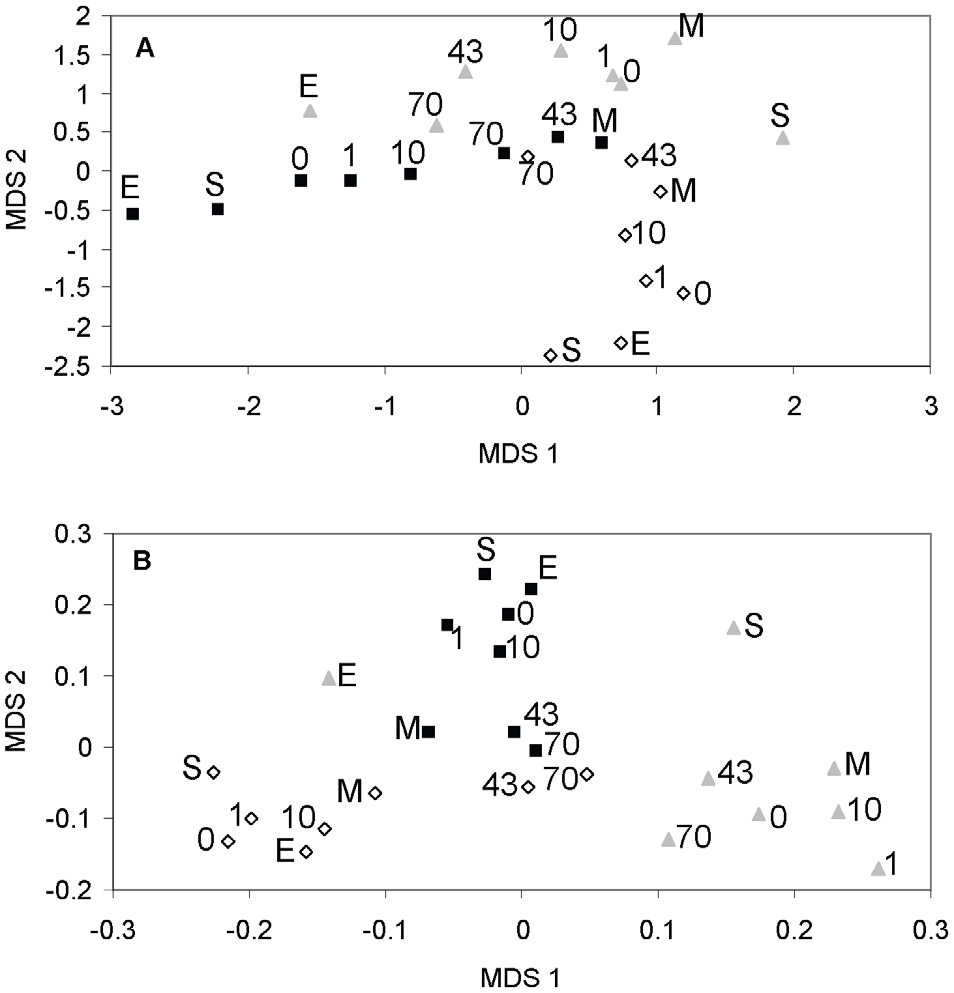
Results from nonmetric multidimensional scaling (NMDS) of BCC. A) shows results using Bray-Curtis index, and B) shows results using Sørensen's index. Each observation is the average from the three experimental replicates. Numbers refer to daily dispersal of standing stock, S = inoculum community at the start of the experiment, E  =  inoculum community at the end of the experiment, and M =  communities from an equal mixture of all three start inoculum communities at the beginning of the experiment receiving no further dispersal. Open diamonds  = oligotrophic lake (Lake Ånnsjön); filled squares  =  humic lake (Lake Fälaren); grey triangles  =  eutrophic lake (Lake Funbosjön).

**Figure 2:**
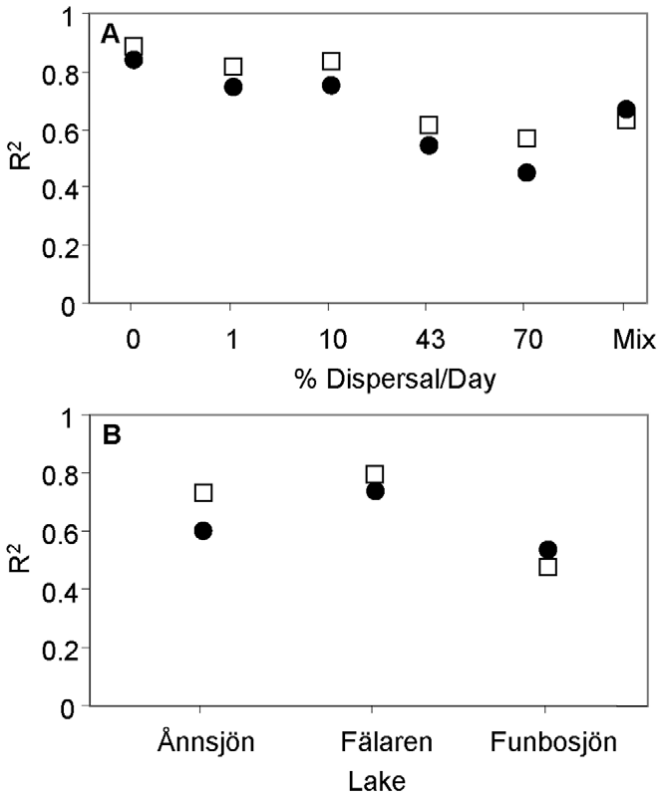
Variation in bacterial community composition (BCC) explained by dispersal treatments and lake water. Variation in Bray-Curtis dissimilarity (filled circles) and Sørensen dissimilarity (open squares) between communities explained (R^2^) by lake water for each dispersal treatment (A), and dispersal treatments for each lake water (B) as assessed by means of PerMANOVAs of the bacterial community composition (BCC). The numbers on the x-axis in A) represent the percentage of biomass replaced per day (i.e. dispersal rates from a metacommunity). M =  the mixed community created at the beginning of the experiment, receiving no further dispersal.

The NMDS analysis showed that communities exposed to the lowest dispersal rates as well as the inoculum/start communities were most dissimilar from another, while communities receiving the highest dispersal (43 and 70% per day) showed greater similarity to each other (Fig. 1). For both similarity measures the PerMANOVA showed treatment effects on BCC of both lake (BC: F_2,45_ = 15, P<0.001, r^2^ = 0.33; Sørensen: F_2,45_ = 26, P<0.001, r^2^ = 0.46) and dispersal (BC: F_5,45_ = 2.9, P<0.001, r^2^ = 0.16; Sørensen: F_5,45_ = 3.3, P<0.001, r^2^ = 0.14). The importance of lake identity for BCC among communities receiving the same dispersal treatment declined with increasing dispersal rate (Fig. 2A). Lake identity explained 50–60%, of the variation in BCC among communities receiving dispersal rates of 43% and 70% per day, respectively, compared to 70–80% among communities at lower dispersal treatments (Fig. 2A). The explanatory power of dispersal rate for variation in BCC among bags within the same lake treatment was greatest in the humic lake (Fälaren) followed by the oligotrophic lake (Ånnsjön) and showing the smallest effect in the eutrophic lake (Funbosjön) (Fig. 2B). The importance of lake environment was somewhat greater when BCC was based on presence-absence similarities compared to when quantitative data was used (Fig. 2A). When the experiment started the communities shared between 60 and 64% of the OTUs above the detection limit. At the two highest dispersal levels, the two lakes being most affected by dispersal shared >75% of the OTUs.

**Figure 3:**
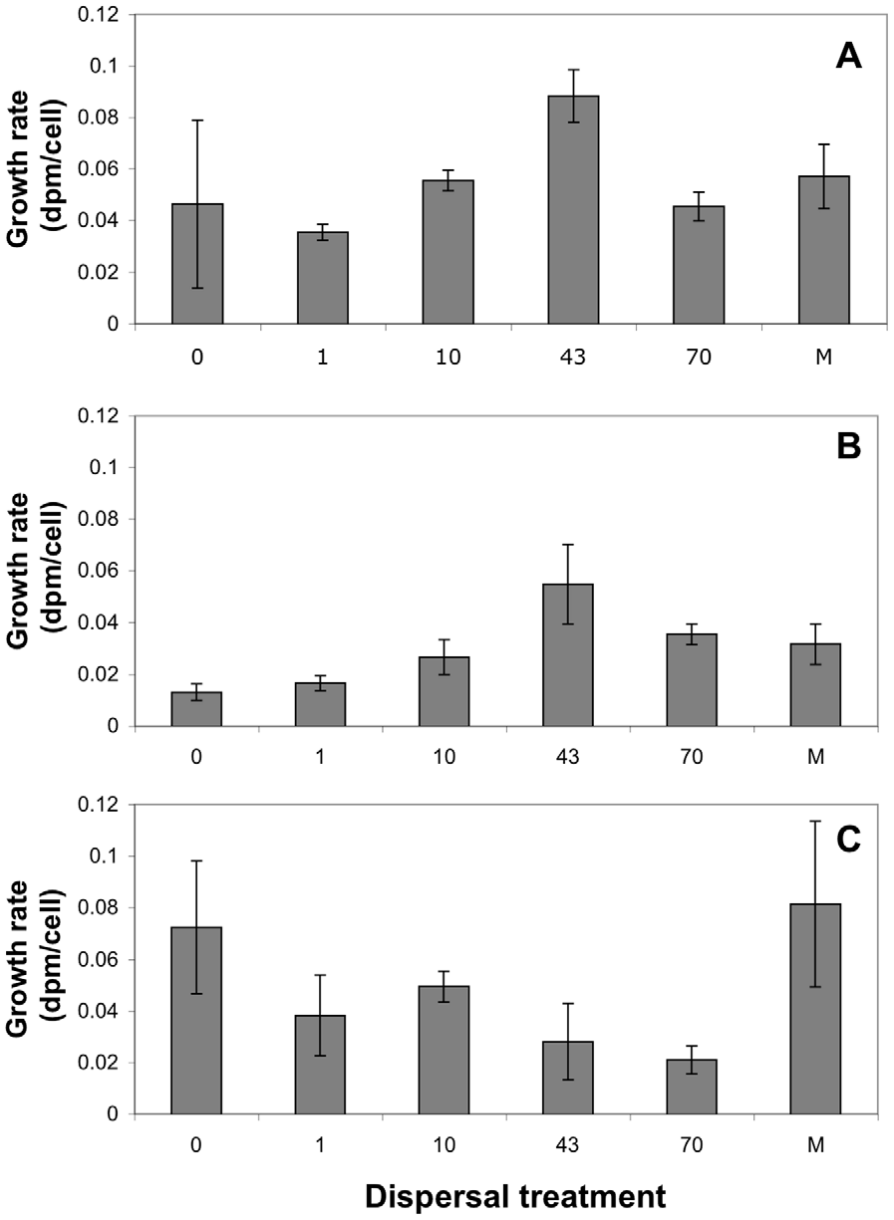
Bacterial growth rates in A) the oligotrophic lake, B) the humic lake, and C) the eutrophic lake. The results shown are means of amount of incorporation of tritiated leucine (disintegration per minute, dpm) per cell and the error bars show standard deviation of triplicate experiments. The numbers on the x-axis represent the percentage of biomass replaced per day (i.e. dispersal rates from a metacommunity). M = the mixed community created at the beginning of the experiment, receiving no further dispersal.

The two-way ANOVA showed that bacterial growth rate (dpm) and growth rate per cell (dpm cell^−1^) differed depending on dispersal treatments (F_5,36_ = 7.7, P<0.001 and F_5,36_ = 5.1, P = 0.001, respectively, N = 54), lakes (F_2,36_ = 12, P<0.001 and F_2,36_ = 14, P<0.001, respectively N = 54) as well as on the interactions between them (F_10,36_ = 7.0, P<0.001 and F_10,36_ = 5.2, P<0.001, respectively, N = 54). In the oligotrophic lake (Ånnsjön) and humic lake (Fälaren) the bacterial growth rate peaked at intermediate dispersal, i.e. at 43% per day (Table 1, Fig. 3 A and B), whereas bacterial growth rate decreased with increasing dispersal in the eutrophic lake (Fig. 3 C). The results were almost identical using the production values directly (dpm) or the dpm per cell (Table 1). In all three lake waters, bacterial community composition was related to growth rate, since one or several NMDS axes (constructed from Bray-Curtis distances) were correlated to bacterial growth rate (Table 2).

**Table 1:** Results from ANOVA of the effect of dispersal treatments on bacterial growth rates.

	Oligotrophic lake	Humic lake	Eutrophic lake
p-value of model	0.017 (<0.001)	<0.001 (<0.001)	0.013 (0.009)
Dispersal treatment	Group	Group	Group
0	B (B)	C (C)	A,B (A,B)
1	B (B)	B,C (B,C)	A,B (A,B)
10	A,B (B)	B,C (B,C)	A,B (A,B)
43	A (A)	A (A)	B (B)
70	B (B)	A,B (A,B)	B (B)
Mix	A,B (A,B)	B,C (B,C)	A (A)

Pair-wise comparisons were done using Tukey's HSD. Treatments designated to the same groups were not significantly different from each other (p>0.05). The results shown here are from analysis of dpm/cell with dpm results within brackets.

**Table 2:** Spearman rank correlation coefficients (Spearman rho) from analysis of the relationship between bacterial community composition (NMDS axes) and growth rate (dpm/cell) in the different lake communities.

	dpm/cell	dpm
Oligotrophic lake (Ånnsjön)	NMDS1: 0.475*	NMDS1: 0.515*
Humic lake (Fälaren)	NMDS1: 0.864***NMDS2: 0.463	NMDS1: 0.847***NMDS2: 0.484*
Eutrophic lake (Funbosjön)	NMDS1: 0.796***	NMDS1: 0.847***

* P<0.05,

*** P<0.001.

Growth rates (dpm) in the negative controls were lower than in all other treatments in the oligotrophic and humic lakes, while in the eutrophic lake two of the negative controls had higher growth rate than the two highest dispersal treatments. In the five (of the nine) negative controls where DNA extraction was possible, >84% of the tRFLP peak area corresponded to peaks also found in the start communities or source communities of the respective lakes. Thus, there appears to have been, in some cases, inflow of viable bacteria from the buckets, either over the dialysis membranes or during handling. This may in some cases have had a marginal impact on the growth rate, and may have affected BCC. Thus, this inflow can have exaggerated the role of the local environment for BCC slightly, especially in low dispersal treatments. An important point is though that there was no abundant “alien” bacterium growing within the bags confounding the results.

By the end of the experiment water colour at 430 nm was similar in the buckets and the dialysis bags receiving the highest dispersal in the humic (0.172 and 0.168, respectively) and in the eutrophic (0.131 and 0.130, respectively) lake. However, in the lake with the clearest water, colour had increased in the highest dispersal treatment by almost 70%, since absorbance was 0.073 in the bucket and 0.124 in the dialysis bag. At the end of the experiment, salt was added in a dialysis bag from the most eutrophic lake (Funbosjön). After 4 h dialysis the salt concentration in the surrounding beaker was 83% of the control beaker. Thus, the diffusion of inorganic ions through the membrane was still efficient at the end of the experiment.

## Discussion

Our results show that both the local environment (i.e. species sorting) and dispersal (i.e. mass effects) affected BCC, which in turn altered bacterial functioning. Low dispersal communities (i.e. 0–10% of standing stock per day) were similar to the start communities of the respective lakes, while the high dispersal communities (i.e. 43 and 70% per day) became more similar to the same treatments from other lakes. The Mixed communities, i.e. identical start communities, kept in the different lake environments but receiving no further dispersal, showed variable results. Thus, high and continuous dispersal rates were necessary to consistently change community composition.

Clearly, the relative importance of species sorting decreased with increasing continuous dispersal rates. However, even at the highest dispersal treatments the local environment (i.e. the lake water) explained a great share of the variation in community composition, and, consequently, the high dispersal communities in the three environments were not identical. Hence, at least parts of the communities were affected by species sorting processes rather than mass effect also at very high dispersal. This conclusion is not likely to change regarding that organic carbon increased with dispersal in one lake. At the opposite, differences in BCC could have been even larger if the environment would have been kept even more similar to that of the bucket.

Results from field studies have shown that not only the immigration rate determines if a community is shaped by mass effect or species sorting. For instance, the position of the habitats of the sink and source communities in the landscape may be of importance [29], suggesting that immigrants may have difficulties to establish themselves in a different habitat. Further, Van der Gucht et al [21] suggested that species sorting should be more efficient in eutrophic waters. In agreement with the latter study, the effect of dispersal on BCC in our experiment was weakest in the eutrophic lake (Fig. 1 and 2). The low growth rates of bacteria in the high continuous dispersal treatments in this lake (Fig. 3) suggest that high concentrations of inorganic nutrients constrain growth rates of many bacterial taxa, and do not simply speed up the local dynamics relative to the regional dynamics as earlier proposed [21]. However, more replication and observational data are needed to clarify if this is a general phenomenon.

In the oligotrophic and the humic lakes the bacterial growth rates peaked at intermediate dispersal levels (43%), i.e. suggesting that also in these lakes variation in BCC imposed by dispersal affected functioning. It should be noted that dispersal increased concentration of organic compounds in the communities from the oligotrophic Lake Ånnsjön, which could have influenced bacterial growth rates. However, since the relationship between dispersal and growth rate was not linear, but rather hump-shaped, this effect did not seem to be a consequence of increased nutrient levels. The close to identical results obtained when using dpm directly or dpm per cell as a measure of growth rate also show that the results were not dependent on changes in cell abundances caused by the dispersal. Thus, we conclude that dispersal appear to have changed growth rate via a change in BCC.

Previous studies investigating the role of microbial dispersal for ecosystem function (local standing biomass) also found highest biomass [7], [8] at intermediate dispersal rates. Both these studies used unsaturated initial communities and the initial increase in functioning was probably due to complementarity. The change in BCC with dispersal in our experiment and especially the increase in the number of shared taxa, suggest that complementarity is a likely explanation also for our results despite that we started with natural communities which may be saturated. For instance, dispersal may have introduced bacteria possessing different resource use capabilities, e.g. means to degrade different organic compounds compared to those in the native communities, or taxa with other life histories, e.g. fast growing taxa. This complementarity is surprising [30] given the high diversity of bacterial communities and presumed high dispersal capacity. However, the dispersal had to be continuous or otherwise these introduced taxa were excluded, as shown by the results from the “Mix” treatment (Fig. 2 and 3). Complementarity is therefore possibly enhancing growth rates also in natural bacterial communities. At higher dispersal rates (70%), growth rate decreased perhaps due to a decrease in the abundance of locally adapted taxa, as expected from mass effects [4], [5], [31], [32].

Therefore, our results show that dispersal can change community function via a change in community composition but that the exact outcome may differ depending on the characteristics of the habitats studied. Future studies therefore need to address how different environments affect the interplay between dispersal, BCC, and functioning. It should also be fruitful to evaluate the possibility that dispersal and establishment may vary among taxa, which would lead to that different populations are affected by different forces. With the future development of techniques for the study of microbial diversity, i.e. sequencing technologies such as 454-sequencing (e.g. [33]), OTU definitions will be more precise compared to the method used here, which will facilitate research on populations rather than communities. Further, the development of sequencing technology makes identification of a greater number of individuals per sample affordable, which may also make it possible to investigate the role of richness and genotypic dissimilarity for function as well as the ability of the migrating bacteria to invade a resident natural community [34], [35].

Future experimenters should also seek for means to investigate more long-term effects of dispersal, which our design did not allow for technical reasons. The start and end communities of Lake Ånnsjön and Lake Fälaren inocula were similar suggesting that community compositions were close to equilibria. In contrast, the start and end communities of the Lake Funbosjön inocula had shifted over the study period (Fig. 1). How this may have affected the relationships between dispersal, BCC and function is not clear. This calls for research on how dispersal may affect BCC and functioning under variable environmental conditions.

Our results together with the observations made by Venail et al. [6] provide one of the first experimental data-sets that contribute to the understanding of under which circumstances regional and local processes should affect local bacterial community composition and bacterial ecosystem functions. These results should be of importance for our understanding of bacterial biogeography as well as for the role of bacterial diversity for ecosystems. In addition, our results show that experimental studies dealing with the importance of substrate and environment for function of bacterial communities may not record the full functional potential if the experimental design hinders dispersal from a metacommunity.

## Supporting Information

Figure S1 There were three buckets (replicates) of lake water from each of the three lakes. The source community (metacommunity) was constructed by equal volumes of water from the three lakes. This source community was then dispersed into the six dialysis bags in each bucket, with different rates, three times per day. The dispersal treatments were replacement of 0, 1, 10, 43 and 70% of the dialysis bags volumes per day. The ”Mix” treatment was a mixture of equal volumes of the start communities from each lake, which thereafter received no further dispersal. All dispersal treatments were made in all buckets.
(TIFF)
