# Supplementary figures and images for: The Importance of Dispersal for Bacterial Community Composition and Functioning
Source: PLoS One. 2011 Oct 6;6(10):e25883. doi: 10.1371/journal.pone.0025883 (PMC3188564; doi:10.1371/journal.pone.0025883)

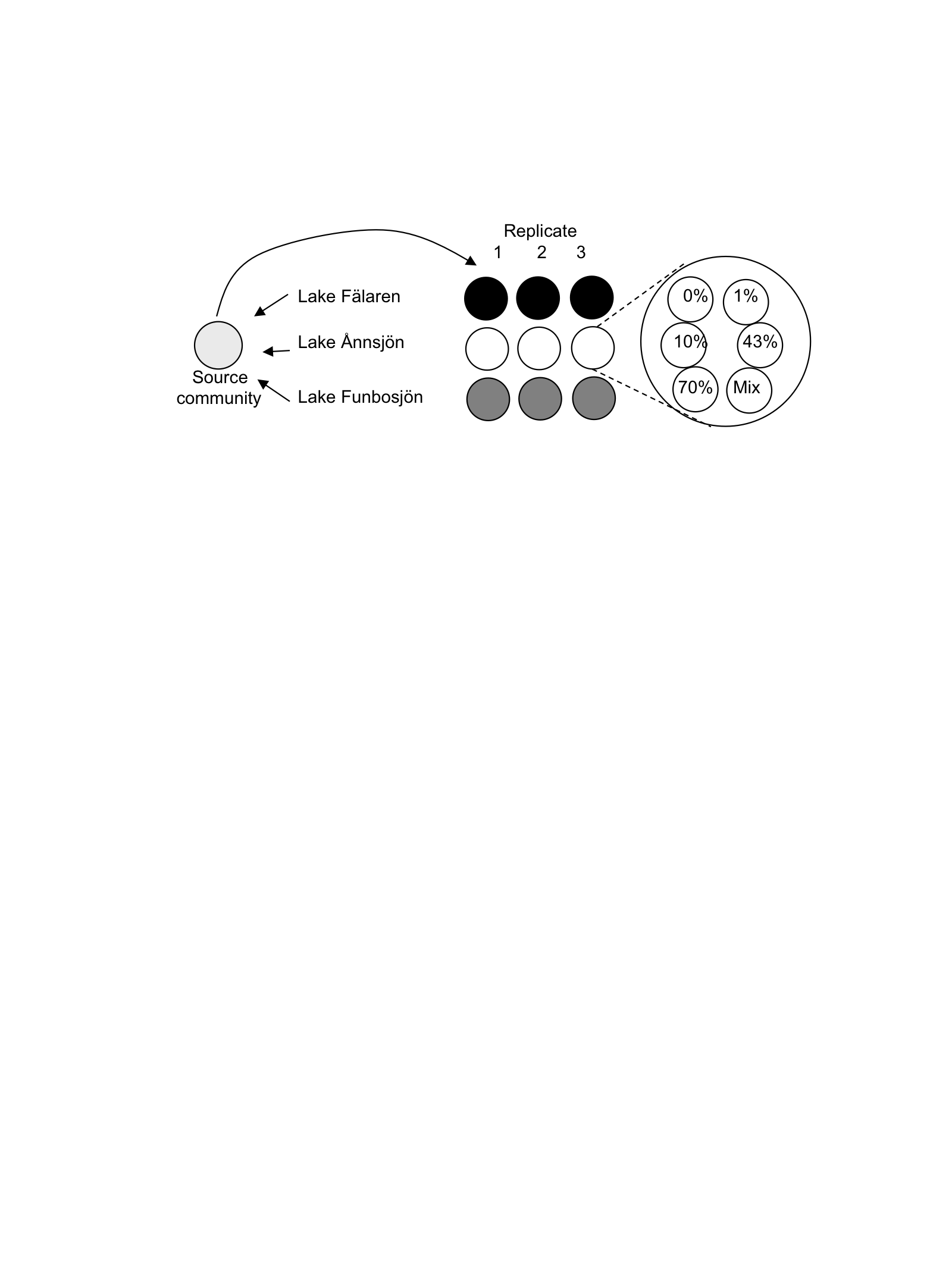

Supplement: Figure S1 — There were three buckets (replicates) of lake water from each of the three lakes. The source community (metacommunity) was constructed by equal volumes of water from the three lakes. This source community was then dispersed into the six dialysis bags in each bucket, with different rates, three times per day. The dispersal treatments were replacement of 0, 1, 10, 43 and 70% of the dialysis bags volumes per day. The ”Mix” treatment was a mixture of equal volumes of the start communities from each lake, which thereafter received no further dispersal. All dispersal treatments were made in all buckets. (TIFF) [file pone.0025883.s001.tiff]
